# Evaporation of inclined water droplets

**DOI:** 10.1038/srep42848

**Published:** 2017-02-16

**Authors:** Jin Young Kim, In Gyu Hwang, Byung Mook Weon

**Affiliations:** 1 Soft Matter Physics Laboratory, School of Advanced Materials Science and Engineering, SKKU Advanced Institute of Nanotechnology (SAINT), Sungkyunkwan University, Suwon 16419, Korea

## Abstract

When a drop is placed on a flat substrate tilted at an inclined angle, it can be deformed by gravity and its initial contact angle divides into front and rear contact angles by inclination. Here we study on evaporation dynamics of a pure water droplet on a flat solid substrate by controlling substrate inclination and measuring mass and volume changes of an evaporating droplet with time. We find that complete evaporation time of an inclined droplet becomes longer as gravitational influence by inclination becomes stronger. The gravity itself does not change the evaporation dynamics directly, whereas the gravity-induced droplet deformation increases the difference between front and rear angles, which quickens the onset of depinning and consequently reduces the contact radius. This result makes the evaporation rate of an inclined droplet to be slow. This finding would be important to improve understanding on evaporation dynamics of inclined droplets.

A droplet is an important element for inkjet printing, painting, and coating technologies that utilize a small volume of colloidal drops^1^,^2^,^3^,^4^,^5^,^6^,^7^,^8^. Evaporation of a droplet is an essential physical process in controlling final deposit patterns of colloids from suspensions, where colloids are uniformly dispersed into a liquid^2^,^9^. A variety of, particularly ring-like, deposit patterns are formed when colloids are left during evaporation on a flat surface from colloidal suspension droplets, as well known as the coffee-ring effect^2^. Evaporation of sessile droplets with a spherical cap shape is well studied theoretically, experimentally, and numerically. For instance, evaporation rates for spherical water droplets are known to depend on contact radius, contact angle, relative humidity, saturated vapor concentration, and vapor diffusivity^1^,^10^.

In practice, raindrops or engineered droplets can be deformed by various reasons such as gravitational influences and surface imperfections^11^,^12^,^13^,^14^. Generally, a droplet is deformed by gravity when its size exceeds a characteristic size, called the capillary length (*l*_*c*_, i.e. ~2.7 mm for pure water^15^). Droplet deformation is determined by competition between surface tension and gravitational force: asphericity increases with drop size. Evaporation of a deformed droplet can be different for that of a spherical droplet, but not clearly understood yet because of geometry complexity of a deformed droplet^3^. A droplet that is slightly larger than *l*_*c*_ and placed on an inclined surface can be asymmetric in shape and its static contact angle divides into front and rear contact angles by inclination. The angle of inclination determines the shape of the droplet. For an inclined droplet, there must exist a larger (*θ*_*f*_ = front) contact angle and a smaller (*θ*_*r*_ = rear) contact angle, which are than its equilibrium contact angle (*θ*_*eq*_) that is radially identical for no inclination^16^,^17^,^18^. Droplet evaporation on inclined surfaces has been not well explored, except for a recent experimental study^3^, demonstrating that the coffee-ring patterns for an inclined surface can be produced differently from that for no inclination: the ring patterns that are deposited on an inclined surface are not uniform radially.

In this work, we experimentally study on how gravity-induced deformation can alter evaporation dynamics of water droplets on inclined surfaces, particularly by modifying pinning-depinning dynamics of droplets that possess front and rear contact angles. On controlling substrate inclination to be 0, 45, 90, 135, and 180 degrees (*ϕ* = 0, *π*/4, *π*/2, 3*π*/4, and *π*, respectively), we measured mass and volume changes of an evaporating droplet with time and analyzed complete evaporation time (lifetime) by varying substrate inclination under gravity. The gravity itself does not change the evaporation dynamics directly^19^,^20^, whereas the gravity-induced deformation increases the difference between front and rear angles, which quickens the onset of depinning and consequently reduces the evaporation rate of an inclined droplet.

## Results

### Evaporation of inclined droplets

**Figure 1:**
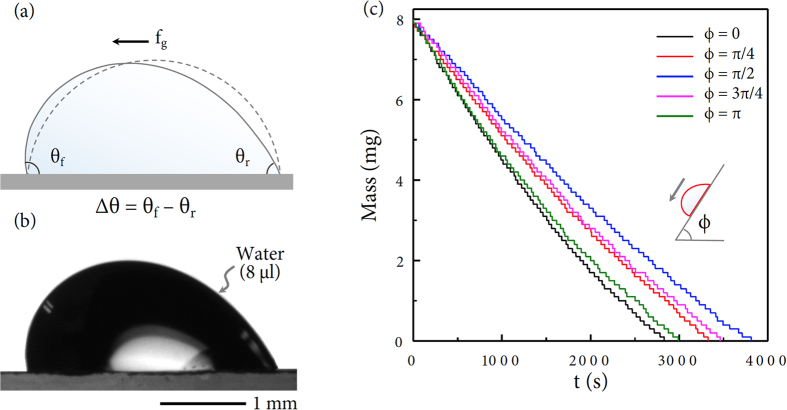
Experimental approach. (**a**) The gravitational force *f*_*g*_ can deform the spherical droplet (depicted as the solid profile) into the inclined asymmetric shape (depicted as the dashed profile) to exhibit front (*θ*_*f*_) and rear (*θ*_*r*_) contact angles. (**b**) The side profile of a pure water droplet at *ϕ* = *π*/2 was taken with the drop shape analyzer. The initial volume to be 8 *μ*l was controlled for comparison in all experiments. (**c**) The mass change during evaporation was monitored with the electronic balance. As the substrate becomes inclined at angle *ϕ*, the mass of the inclined droplet monotonically decreases with time during evaporation. Interestingly the droplet at *ϕ* = *π*/2 has the longest lifetime. This dataset demonstrates that the evaporation rate of the inclined droplet varies with the inclined angle of the substrate.

We controlled experimental conditions to explore gravitational influence on evaporation dynamics of an inclined droplet. A pure water droplet with an initial 8 *μ*l volume was placed on a clean glass substrate. A nearly spherical water droplet at no inclination was then influenced by gravity to exhibit front (*θ*_*f*_) and rear contact angles (*θ*_*r*_) by tilting the substrate, as illustrated in Fig. 1(a). The difference of front and rear contact angles Δ*θ*, called the contact angle hysteresis, is dependent upon the gravitational force *f*_*g*_. Here gravity slightly deforms the shape of the droplet, as demonstrated for an inclined droplet at angle *ϕ* = *π*/2 in Fig. 1(b). On tilting the substrate, the mass and the volume of the droplet could be measured with the drop shape analyzer and the electronic balance. The measurement was repeated for individual droplets with inclined angles, as representatively depicted in Fig. 1(c). For each inclined angle, a droplet was selected for demonstration because its lifetime is close to the average lifetime. Here, the lifetime of the inclined droplet varies with the inclined angle of the substrate.

### Lifetime of inclined droplets

**Figure 2:**
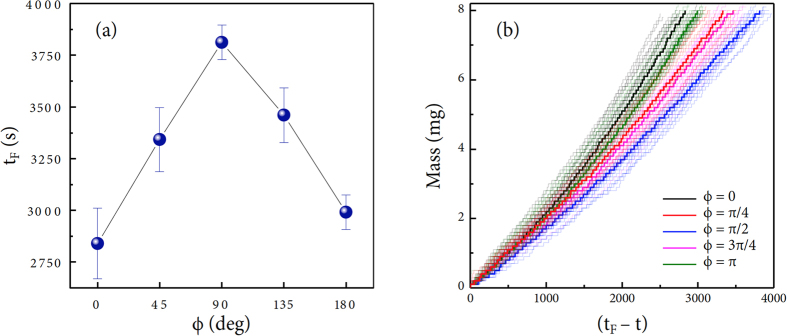
Lifetime and evaporation dynamics. (**a**) The droplet lifetime *t*_*F*_ until complete evaporation for 8-*μ*l-volume pure water droplets at different inclined angles *ϕ*. The original lifetime value for the individual inclined droplet was measured at time for the mass to be zero, as shown in (**b**). The error bars were taken from quite a number of datasets (ranging from 17~24 droplets for each angles). (**b**) The merged mass change data in order of *ϕ* = 0, *π*/4, *π*/2, 3*π*/4, and *π*, respectively. It is noteworthy that the lifetime at *π*/2 is the longest. This result demonstrates that the evaporation behavior of the inclined droplet is strongly dependent upon the substrate inclination and hence the gravitational influence. The bold lines represent selected droplets for each inclined angle, because their lifetime is close to the average lifetime.

The droplet lifetime *t*_*F*_ until complete evaporation for 8-*μ*l-volume pure water droplets at different inclined angles is summarized in Fig. 2(a), taken from the mass evolution data with the electronic balance. The each lifetime *t*_*F*_ was measured at time for the mass to be zero, as illustrated in Fig. 2(b). The statistics for the lifetime is described in the error bars, taken from quite a number of datasets (ranging from 17~24 droplets) in order of *ϕ* = 0, *π*/4, *π*/2, 3*π*/4, and *π*, respectively. It is noteworthy that the lifetime of the inclined droplet changes with the inclined angle of the substrate: particularly, the lifetime at *ϕ* = *π*/2 is the longest, corresponding to the strongest gravitational influence on the vertical substrate. The force acting the droplet on the inclined surface *f* = *f*_*g*_ sin *ϕ* where *f*_*g*_ = *mg* (with *m* = mass and *g* = gravitational acceleration)^11^,^17^,^18^ is maximized as the inclined angle reaches the right angle (*ϕ* → *π*/2, sin *ϕ* → 1). This result implies that the lifetime of the inclined droplet becomes longer as the gravitational influence becomes stronger. High similarity in the lifetime appears between *ϕ* = 0 and *π* as well as between *ϕ* = *π*/4 and 3*π*/4, because of the identical gravitational influences (sin 0 = sin *π* and sin(*π*/4) = sin(3*π*/4)). Interestingly, the lifetime of the droplet beneath the substrate for *ϕ* = 3*π*/4 and *π* is slightly longer than that on the substrate for *ϕ* = 0 and *π*. The gravitational effect on the lifetime diminishes at the late stage of evaporation because the droplet size becomes smaller than the capillary length^15^. These lifetime results demonstrate that the evaporation behavior of the inclined droplet is strongly dependent upon the substrate inclination unless the droplet size is smaller than the capillary length.

### Pinning-depinning transition

**Figure 3:**
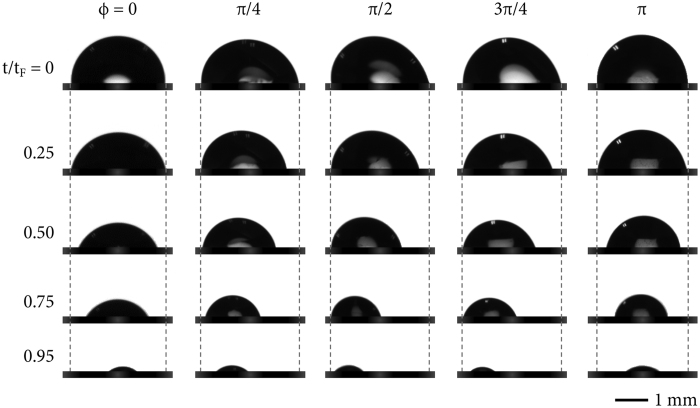
Pinning-depinning transition. The shape and the volume changes of the droplet with the substrate inclination were taken from the side profile images of the droplet by utilizing the drop shape analyzer. The time scale is normalized by dividing the evaporation time by the lifetime *t*/*t*_*F*_ for comparison.

The shape and the volume changes of the droplet with the substrate inclination are shown in Fig. 3, taken from the side profile images of the droplet by utilizing the drop shape analyzer. The time scale is normalized by dividing the evaporation time by the lifetime (*t*/*t*_*F*_). Interestingly, for an 8-*μ*l-volume pure water droplet at *ϕ* = 0 (no inclination), the initial contact line is pinned until *t*/*t*_*F*_ = 0.25 and then depinned after *t*/*t*_*F*_ = 0.5: it eventually almost disappears at *t*/*t*_*F*_ = 0.95. This behavior at *ϕ* = 0 similarly occurs for the droplet at *ϕ* = *π*. Since the substrate inclination changes the shape of the droplet, the initially nonspherical droplets are found at *ϕ* = *π*/4, *π*/2, and 3*π*/4. As the inclined angle approaches to the right angle, the force acting the droplet increases and deforms the shape of the droplet. The gravitational influence alters the pinning-depinning behavior of the inclined droplet^21^. As shown in Fig. 3, the depinning phenomenon significantly takes place at rear contact angles (at the right side), rather than front contact angles (at the left side), as demonstrated as the apparent leftward shift of the droplet, implying that the rear contact angle is able to be depinned first by inclination under gravity.

### Inverse proportion of lifetime and pinning-depinning transition

**Figure 4:**
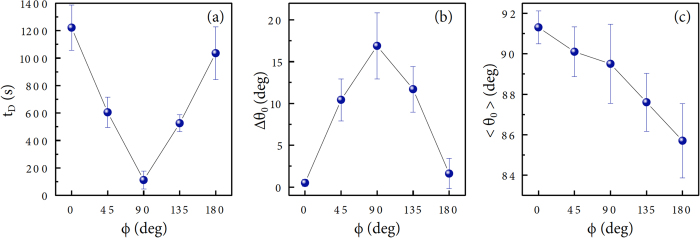
Correlation between depinning occurrence and contact angles. (**a**) The onset time of depinning *t*_*D*_ for the inclined droplet shows an inverse proportion to the droplet lifetime *t*_*F*_ in Fig. 2(a). (**b**) The initial contact angle hysteresis Δ*θ*_0_ shows a normal proportion to the droplet lifetime. (**c**) The average initial contact angle 〈*θ*_0_〉 for the inclined droplet, taken by averaging *θ*_*f*_ and *θ*_*r*_ at the initial time, has no relevance with the lifetime, except showing a gradual decrease with the substrate inclination *ϕ*.

To gain additional insight into the pinning-depinning transition by varying the substrate inclination, we measured the onset time of depinning *t*_*D*_ for the inclined droplet in Fig. 4(a), showing how rapidly the pinning-depinning transition takes place. Here the observed *t*_*D*_ values clearly vary with the inclined angles: particularly, the inclination dependence of *t*_*D*_ in Fig. 3(a) is in inverse proportion to that of *t*_*F*_ in Fig. 2(a). In addition, the inclination affects the initial difference of front and rear contact angles, Δ*θ*_0_, [Fig. 4(b)] particularly in the reverse direction with *t*_*D*_ [Fig. 4(a)]. The inverse proportion in *t*_*F*_ and *t*_*D*_ as well as the longest *t*_*F*_, the shortest *t*_*D*_, and the largest Δ*θ*_0_ at *ϕ* = *π*/2 would be associated with the maximization of the gravitational influence by inclination.

## Discussion

To explain the inverse proportion between the lifetime [Fig. 2(a)] and the pinning-depinning transition [Fig. 4(a)], we need to find out better explanation to connect the droplet geometry with the evaporation mode. However it is yet difficult to establish a theoretical model for the evaporation rate of the inclined droplet because of the complexity in the shape of the droplet and the pinning-depinning transition.

According to the general evaporation equation, the evaporation rate of the spherical sessile droplet depends on the initial contact angle *θ*_0_ and the lifetime of the spherical droplet is nearly deterministic as a function of *θ*_0_^1^,^4^,^6^,^10^. Contrary to the spherical droplet, the average initial contact angle 〈*θ*_0_〉 for the inclined droplet, taken by averaging *θ*_*f*_ and *θ*_*r*_ at the initial time, has no relevance with the inclination dependences of the lifetime [Fig. 2(a)], the pinning-depinning transition [Fig. 4(a)], and the initial angle difference [Fig. 4(b)]. Interestingly the 〈*θ*_0_〉 value gradually decreases with the inclined angle, as can be seen in Fig. 4(c). The gradual decrease of the average initial angle 〈*θ*_0_〉 with the inclined angle is due to the gravity-induced sagging effect^22^ which is minimized at *ϕ* = 0 and maximized at *ϕ* = *π* (see a similar observation^23^).

**Figure 5:**
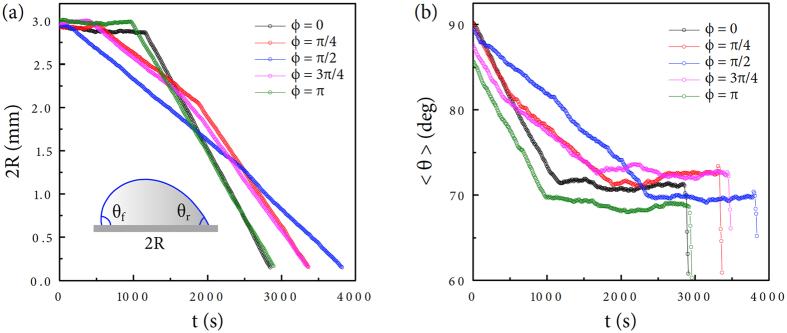
Complexity in evaporation dynamics. (**a**,**b**) The contact-line diameter 2*R* and the average contact angle 〈*θ*〉 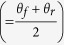 were measured from the movies for Fig. 3. The combined evaporation mode appears for the evaporation dynamics of the inclined droplet at *ϕ* = 0 and *π* and the mixed evaporation mode at *ϕ* = *π*/4, *π*/2, and 3*π*/4.

The evaporation rate of the inclined droplet shall be proportional to the contact radius as −*dv*/*dt* ∝ 2*πR*^1^,^4^,^6^,^10^. This proportion is associated with the diffusion-limited evaporation by which the highest evaporative flux exists at the contact line^3^. The pinning-depinning transition is an important phenomenon that takes place at the contact line and that influences the contact radius, which is crucial to determine the evaporation lifetime^24^. The contact-line diameter 2*R* and the average contact angle 〈*θ*〉 were measured and demonstrated in Fig. 5(a,b) from the representative droplets of Fig. 1(c). Here the evaporating droplet at *ϕ* = *π*/4, *π*/2, and 3*π*/4 exhibits a mixed evaporation mode, where the contact radius *R* and the contact angle *θ* simultaneously vary with time^24^. The evaporation mode found for *ϕ* = 0 and *π* shows a typical combined mode, where the contact line is initially pinned and in turn depinned after the pinning-depinning transition time. The occurrence of the combined or the mixed mode is attributed to the complexity in the pinning-depinning transition with the substrate inclination.

A recent work has developed a theoretical master curve for the droplet lifetime, which is available for the pinning, the receding, and the combined modes^5^. Particularly for a droplet with initial contact angles of *π*/2, consistent with our droplets, the droplet lifetime at the combined mode is almost identical to the lifetime at the single receding mode^5^. For the receding mode based on the spherical cap geometry, the evaporation rate is expressed as 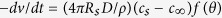 where *R*_*s*_ is the radius of the sphere, *D* is the diffusion coefficient, *c*_*s*_ is the vapor concentration at the liquid surface, *c*_∞_ is the vapor concentration at infinite distance, *ρ* is the liquid density, and *f*(*θ*) is a function of the contact angle of the droplet^25^. The exact form of *f*(*θ*) is different to researchers (Picknett and Bexon^26^, Rowan *et al*.^27^, and Bourgés-Monnier and Shanahan^28^).

**Figure 6:**
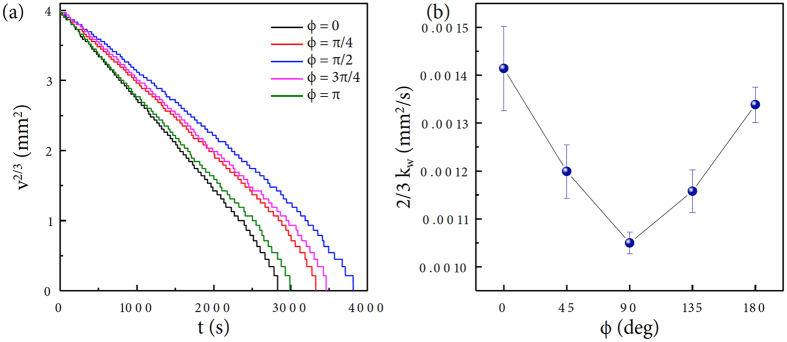
Empirical interpretation of evaporation rates. (**a**) The volume evolution data taken in Fig. 1(c) are rescaled to be *v*^2/3^ versus *t*. Here, the nearly linear relation appears as 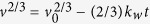, where *k*_*w*_ is an experimental constant. (**b**) The evaporation rate constant (2/3) *k*_*w*_ taken from all the tested droplets has an inverse proportion to the lifetime tendency in Fig. 2(a).

The volume change with time for the receding mode can be simplified as 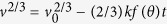 where *v*_0_ is the initial volume and *k* is a constant independent of the evaporation time^1^,^25^. Despite the complexity in the droplet geometry and the pinning-depinning transition, we adopted the evaporation equation from the spherical cap geometry to describe the volume change of the inclined droplet as


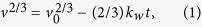


where *k*_*w*_ is an experimental constant^29^. We converted the mass evolution data from Figs 1(c)–6(a) and estimated the slope to obtain the (2/3) *k*_*w*_ value: this evaluation was repeated for all the tested droplets and summarized in Fig. 6(b). The measured (2/3) *k*_*w*_ value can be used to evaluate how much gravity-induced droplet deformation changes the evaporation rate of the inclined droplet. This *v*^2/3^ versus *t* relation indicates *v* → 0 as *t* → *t*_*f*_ (the complete evaporation time)^29^. The evaporation rate is taken as


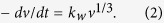


The evaporation rate would be proportional to the contact radius *R* of the inclined droplet. Here, −*dv*/*dt* ∝ *v*^1/3^ is mathematically consistent to −*dv*/*dt* ∝ 2*πR* because *R* ∝ *v*^1/3^.

The contact-line dependence of the evaporation rate as −*dv*/*dt* ∝ 2*πR* implies that the rapid pinning-depinning transition by inclination under gravity would be responsible for the long lifetime for the vertical substrate condition, because it is able to shrink the contact radius *R*, inevitably inducing the decrease of the evaporation rate. Consequently, the inverse proportion between the lifetime [Fig. 2(a)] and the depinning onset time [Fig. 4(a)] with the substrate inclination can be explained roughly by the contact-line dependence of the evaporation rate. For the inclined droplet, the gravity-induced substrate inclination would facilitate the pinning-depinning transition and thus can make the droplet to slowly evaporate.

Considering the possible onset of convection in the atmosphere which would enhance the evaporation^8^,^30^,^31^, one may expect the onset of convection on the vertical substrate and the shorter lifetime than on the horizontal substrate. However, our experimental results clearly show that the lifetime of an evaporating droplet on a tilted substrate is controlled by the depinning dynamics of the contact line and not by the onset of convection. The depinning force *f*_*d*_ generates from the unbalanced Young’s force as 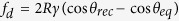 where *θ*_*rec*_ and *θ*_*eq*_ are the receding and the equilibrium contact angles and *γ* is the surface tension of water^32^,^33^,^34^,^35^,^36^,^37^,^38^,^39^. Here the gravity force would reinforce the depinning force as *f*_*d*_ + *f*_*g*_ sin *ϕ*, which can facilitate the pinning-depinning transition time *t*_*D*,*ϕ*_ in order of *t*_*D*,*π*/2_, 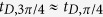, and 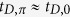 [Fig. 4(a)]. The decreased 〈*θ*_0_〉 with the inclined angle [Fig. 4(c)] would be responsible for the different pinning-depinning time as *t*_*D*,0_ > *t*_*D*,*π*_ or *t*_*D*,*π*/4_ > *t*_*D*,3*π*/4_ [Fig. 4(a)], because the initial rear contact angle is close to the receding contact angle under gravity.

Finally, we state that there would be a simple qualitative explanation for the experiments. Initially, the inclined droplet would be roughly at the marginal equilibrium: the front angle slightly below the advancing contact angle and rear angle slightly above the receding contact angle. As the contact line is pinned, the effect of evaporation at short time is to decrease both contact angles. The front angle stays within the advancing and the receding contact angles and the contact line at the front of the droplet would stay pinned. On the other hand, at some time given by the initial conditions and the rate of evaporation, the rear angle of the droplet becomes smaller than the receding contact angle and the contact line recedes. At this moment, the total evaporation rate decreases compared to that for the horizontal substrate because the radius of the wetted area is reduced. When both contact angles are below the receding angle, both contact lines would recede. Ultimately, for small droplets (*R* < *l*_*c*_), there would be no effect of inclination by gravity. Presumably, the pinning-depinning time would be a function of how far from the receding contact angle is the rear angle of the inclined droplet in the beginning of the evaporation process.

To conclude, we presented an experimental study on how gravity-induced deformation can alter evaporation dynamics of droplets on inclined surfaces, particularly by modifying pinning-depinning dynamics of droplets that possess front and rear contact angles. Practically, raindrops or engineered droplets can be deformed by various reasons such as gravitational influences and surface imperfections. Droplet deformation and pinning-depinning transition would significantly contribute to the complexity of the droplet evaporation dynamics. Our finding would give better insight for explanation and prediction of the evaporation dynamics of the inclined droplet.

## Methods

### Evaporation experiments

All experiments were conducted at 25 ± 2 °C and 30 ± 2% relative humidity. Pure (deionized) water was obtained from DI water system (ELGA) and the initial volume of the water droplet was controlled to be 8 *μ*l for all experiments. For the mass evaluation, the electronic mass balance (EX224G, Ohaus) with 0.1 mg readability was utilized. Prior to evaporation, a 8-*μ*l-volume pure water droplet was delivered from a micro pipette on the substrate. All droplets were gently put on the horizontal substrate before tilting the substrate to the correct inclination. The mass of the droplet was measured automatically for every 1 second during evaporation. For the shape and the volume evaluation, the drop shape analyzer (DSA25, Krüss) with back light and a CCD camera was adopted. The acquisition of the image was taken automatically for every 10 seconds. The data for radius and contact angle of droplets were acquired in real time from drop shape images with the general conic section method (tangent method 1, Krüss DSA25).

### Substrate preparation

To rule out the substrate variability, the glass wafer (i-Nexus) was immersed into 1 wt% fluoroalkylsilane (FAS-17, purchased from Tokyo Chemical Industry) ethanol solution for 24 hours, dried at 120 °C for 2 hours, and sonicated in ethanol for 10 minutes. Then, the initial contact angle of the substrate was controlled to be 91 ± 1 degrees for the 8-*μ*l-volume pure water droplet.

## Additional Information

**How to cite this article**: Kim, J. Y. *et al*. Evaporation of inclined water droplets. *Sci. Rep.* **7**, 42848; doi: 10.1038/srep42848 (2017).

**Publisher's note:** Springer Nature remains neutral with regard to jurisdictional claims in published maps and institutional affiliations.
